# Predictors of Readiness to Quit Stages and Intention to Quit Cigarette Smoking in 2 and 6 Months in Lebanon

**Published:** 2017-05-11

**Authors:** Nelly Layoun, Souheil Hallit, Mirna Waked, Zeina Aoun Bacha, Isabelle Godin, Michele Dramaix, Pascale Salameh

**Affiliations:** ^1^ Research Center in Epidemiology, Biostatistics and Clinical Research, School of Public Health, Free University of Brussels, Brussels, Belgium; ^2^ Doctoral School of Sciences and Technologies, Lebanese University, Beirut, Lebanon; ^3^ School of Pharmacy, Lebanese University, Beirut, Lebanon; ^4^ Faculty of Pharmacy, Saint Joseph University, Beirut, Lebanon; ^5^ Faculty of Pharmacy, Holy Spirit University, Kaslik, Lebanon; ^6^ Occupational Health Environment Research Team, Population Health Research Center, Bordeaux University, Bordeaux, France; ^7^ Research Department, Psychiatric Hospital of the Cross, Jal Eddib, Lebanon; ^8^ Department of Pulmonology, St George Hospital University Medical Center; Faculty of Medicine, Balamand University, Lebanon; ^9^ Department of Pulmonary and Critical Care Medicine, Hotel-Dieu de France, Beirut, Lebanon; ^10^ Faculty of Medicine, Lebanese University, Beirut, Lebanon

**Keywords:** Cigarette Smoking, Motivation, Readiness potential, Smoking Cessation

## Abstract

**Background:** We aimed at examining quitting behaviors among Lebanese cigarette smokers in order to clarify characteristics of adults who were more likely to intend to quit smoking.

**Study design:** A cross-sectional study.

**Methods:** This study was conducted between March 2014 and March 2015, involving 382 patients randomly chosen from 5 outpatient clinics in 5 hospitals in Lebanon. A standardized questionnaire was completed including socio-demographic characteristics, smoking behavior, chronic respiratory symptoms, Fagerstrom scale, Mondor scale, packaging perception, quitting behavior and readiness to quit ladder.

**Results:** 40.8% of participants reported having higher stages of readiness to quit while 33% and 7.9% of them intended to quit in 2 and 6 months later, respectively. Higher stages of readiness to quit were associated with high motivation to quit smoking (ORa=1.98; *P*=0.007), chronic wheezing and real quit attempt duration of ≥ 1 month (ORa=2.35, *P*=0.020 and ORa=2.15, *P*=0.003, respectively). Highly motivated smokers (ORa=1.83, *P*=0.040), who would have changed their favorite pack due to the graphical warnings (ORa=2.11, *P*=0.010) and who had past quit attempt (ORa=4.39, *P*<0.001) had more intention to quit in 2 months. Having past quit attempts would increase the intention to quit in 6 months by 7.48 times (ORa=7.48, *P*=0.007).

**Conclusions:** Significantly higher intentions to quit cigarette smoking were associated with a higher motivation and influenced by shocking images and health related warnings on tobacco boxes. We hope our results will initiate public health educational programs and interventions to surge the intention to quit cigarette smoking as the first step of quitting.

## Introduction

Smoking remains a primary public health concern worldwide despite its decreasing frequency in developed counties ^1^. It is the primary avoidable reason of chronic morbidity and mortality ^2^. According to WHO report in 2015, on the global tobacco epidemic, the smoking frequency in Lebanon reached 36.2% ^1^, the highest in the Middle East and North Africa region ^3^. Cigarette smoking is the principal risk factor for the damaging consequences on the respiratory and cardiovascular systems ^4^, acute exacerbations of respiratory illness, and associated morbidity as well as mortality ^4^.

The stages of change (SOC) theory has been used in many interactive programs to assist with the smoking cessation process^5^. It considers that smoking cessation is a procedure program consisting of five motivational phases^6^, each representing a changed chronological and motivational feature of behavioral alteration and adjustment ^7^. The first three out of five stages define the individuals’ readiness to quit smoking. These phases consist of: a. pre-contemplation (no intention to quit); b. contemplation (intention to quit smoking during the subsequent 6 months); and c. preparation (determination to quit smoking within the subsequent 30 days). Individuals are considered to be in the action stage once they quit smoking for 6 months and in the maintenance stage if they quit for a period of 6 months to 5 years. However, if they stopped smoking for more than 5 yr, they are considered to be at the termination stage. The challenges to quitting smoking remain at 1- and 6-month follow-up ^7^.

Moreover, motivation to quit is an important concept in the smoking cessation procedure ^8^; although studies reveal a lack of compromise on how such “motivation” is well-defined and measured^9^. In the general public, high motivation levels as stated by smokers who have high determination to quit, have been correlated with the search for cessation support^10^. Hence, multiple factors were associated with the person’s readiness to quit smoking. Many smokers who try to quit cite a desire to improve their health as the main reason^11^. Furthermore, demographic factors (gender, age, marital status, income, and education) were studied to compare between smokers who had quit attempts or not, as well as between successful and unsuccessful quitting attempts^12^.

In spite of an increased frequency of smoking in Middle East region, few researches were done on the intention to quit smoking in Arab countries. Therefore, the main objectives of this study were to examine quitting behaviors among Lebanese cigarette smokers in order to clarify characteristics of adults who were more likely to intend to quit smoking, better define quitting behaviors among these smokers and ultimately establish effective interventions for cigarette adult smokers.

## Methods

### Study design and ethics

this prospective study was conducted between March 2014 and March 2015 in 5 outpatient clinics in 5 hospitals in Lebanon, randomly enrolling patients to enter the study. The Lebanese University approved the study as an observational study; respected participants’ autonomy and confidentiality are observed; and principles of the Declaration of Helsinki are followed.

### Participants

Subjects were randomly chosen to complete a standardized questionnaire in the waiting rooms of respiratory outpatient clinics and of a smoking cessation center located in one hospital in Beirut; Eligible participants were current exclusive adult cigarette smokers defined as “currently smoking ≥ 1 cigarette per day” and visiting the clinic for an ordinary check-up or for an acute respiratory disease including pneumonia, bronchitis or a chronic obstructive pulmonary disease. Patients seeking advice for a smoking cessation program were also eligible to participate. Participants were interviewed in Arabic by a health care provider trained to use standardized questionnaires. This study design has been previously described in the Italian population^13, 14^.

### Sample Size

The sample size was calculated using the following formula of Charan and Biswas ^15^: $n=\frac{z^{2}.p(1-p)}{d^{2}}$ where Z=1.96 (when the confidence interval is 95%), p= expected proportion in population based on other studies, and d=absolute error or precision (5%). Based on the report of the Centers for Disease Control and Prevention report done in the United States where 68.8% of current adult smokers want to completely stop smoking ^16^, we calculated a minimal sample size of 330 patients required to allow for adequate power for bivariate and multivariate analysis to be carried out on several factors and to give a 95% probability of measuring the prevalence of intention to quit with 5% accuracy.

### Tools and variables

The pretested questionnaire from the standardized questionnaire of the American Thoracic Society was given to all participants ^17^. It was adapted to local Arabic language (the native language in Lebanon); details about the translation process were presented in another study ^18^. Patients needed an average of 20 min to complete the questionnaire. Socio-demographic characteristics, including age categorized into ≤45 yr and >45 yr, gender, region categorized into Beirut, Mount and North, employment status divided into employed, unemployed and never employed, educational level divided into low education (illiterate, primary, complementary and secondary levels) versus high education (university level) and the marital status categorized into married versus single status (single, divorced or widowed) were assessed.

Concerning the smoking behavior, we asked about the cigarette smoking status, the number of cigarettes smoked per day categorized into 1 to 9, 10-25 and >25 cigarettes per day^14^, the family smoking status categorized into ≤1 person who smoked in the same house versus >1 person, if the patient smoked indoor, the number of smokers at work categorized into ≤1 smokers or >1 person and submission to tobacco smoking at work. The age of cigarette smoking onset was categorized into 10 to 14, 15 to 17 and ≥18 yr^19^.

The presence of chronic respiratory symptoms was defined as an affirmative answer to multiple questions; Chronic respiratory problems were assessed using the following definitions using the International Study of Asthma and Allergies in Childhood (ISAAC) questionnaire ^20^: “Chronic respiratory disease diagnosed by a doctor, reported a chronic cough, reported chronic phlegm, chronic bronchitis defined as a cough and phlegm for >3 months per year since 2 years, cough and phlegm for >3 weeks and chronic wheezing”. More details about the chronic symptoms were presented already ^13^.

The cigarette smoking dependence status was measured via the Fagerstrom scale. Scores were categorized into 1-4 “low dependency” and ≥5 “high dependency” ^14^. The motivation to quit smoking was measured using the Mondor scale; scores were categorized into ≤12 reflecting a low motivation to quit and >12 reflecting a high motivation to quit ^21^.

In order to assess the packaging perception, two different types of warnings were shown to the smokers during the interview: Only text (current warning used in Lebanon) versus pictorial “shocking” warnings (i.e., diseased lungs, throat cancer and rotting teeth). To quantify the effect of the warning, two questions were asked; details about the textual and pictorial effect on the quitting behavior were presented already^13^.

Quit attempts were assessed by asking smokers, “how many times during the last year have you stopped smoked for 1 day (24 hours) or longer?” Responses were categorized into zero quit attempts and ≥1 quit attempt. Real quit attempts durations were assessed by asking smokers: “how long have you been staying without smoking any cigarette?” Answers were categorized into <1 month and ≥1 month. Intention to seriously quit cigarette smoking in 2 months “no/yes. Intention to seriously quit cigarette smoking in 6 months “no/yes.

We assessed the motivation to quit smoking by using the readiness to quit ladder. The Ladder is a continuous measure of motivation to change smoking behavior that uses a 10-point scale with responses ranging from 1 = "I have decided to continue smoking" to 10 = "I have already quit smoking." Validity studies have demonstrated that the Ladder is associated with cognitive and behavioral indices of readiness to consider smoking cessation (e.g., intention to quit, nicotine dependence) and performs as well or better than the staging algorithm in predicting smoking rate, quit attempts and cessation ^22-24^.

We divided the scale into 2 subgroups, the low-motivated one including the pre-contemplation (not thinking about quitting) phases and the high-motivated one containing the “contemplation (thinking about quitting but not ready to quit), preparation (getting ready to quit), action (quitting) and maintenance (remaining a non-smoker) phases.

### Statistical analysis

Data analysis was performed on SPSS software version 23 (Chicago, IL, USA). Categorical data were shown as absolute frequencies and percentages. Two sided statistical tests were used; Chi-2 test or the Fisher's exact test for dichotomous or multinomial qualitative variables.

Regarding multivariate analysis, 3 logistic regressions were performed, taking into account the variables in the bivariate analysis that showed a *P*-value < 0.2 ^25^; potential confounders may be eliminated only if *P*>0.2, in order to protect against residual confounding ^25^. Furthermore, we considered the readiness to quit (low versus high motivation to quit), the intention to quit smoking in 2 months (Yes/No) and the intention to quit smoking in 6 months (Yes/No) as dependent variables respectively. The statistical significance was set at a *P*-value< 0.05.

## Results

We calculated the reliability of each scale to assess the quality of our data. We obtained high Cronbach alphas for all scales as follows: Mondor scale (0.757) and Fagerstrom scale (0.789).

In total, data was collected from 382 cigarette smokers with a response rate of 88%. Table 1 summarizes the socio-demographic characteristics of those cigarette smokers. Sixty one percent of the participants were males; more than half were more than 45 yr old.

**Table 1:** Sociodemographic characteristics of cigarette smokers in Lebanon.

**Factor**	**Cigarette (n=382)**	
	**Number**	**Percent**
Gender		
Male	233	61.0
Female	149	39.0
Age group		
≤45 yr	188	49.2
˃45 yr	194	50.8
Marital status		
Married	253	66.2
Single	129	33.8
Education		
Low	215	56.3
High	167	43.7
Work situation		
Employed	280	73.3
Unemployed	39	10.2
Never employed	63	16.5
Residence		
Beirut	108	28.3
Mount Lebanon	160	41.9
North	114	29.8
Number of smokers in the family		
≤ 1 person	185	48.4
> 1 person	197	51.6
Number of persons smoking inside the house		
No	137	35.9
Yes	245	64.1
Number of persons smoking at work		
≤ 1 person	210	55.0
> 1 person	172	45.0
Submission to smoke at work		
No	227	59.4
Yes	155	40.6

The first bivariable analysis was conducted taking the readiness to quit as the dependent variable. The results showed that smokers having chronic wheezing were significantly more motivated to quit (17.9% versus 9.5%, *P*=0.020), same as people with high motivation as shown by the Mondor scale score (58.3% versus 34.3%, *P*<0.001). Furthermore, smokers with no curiosity to ask a specialist to help them to quit smoking were less motivated to stop smoking (*P*<0.001), while smokers who have ever stopped smoking for at least one month due to the textual warning already implemented on cigarette packages and who considered it is very important to report health warnings on packs were highly motivated to quit with (30.1% versus 16.2%, *P*<0.001) and (60.3% versus 35.2%) respectively. In addition, smokers who have ≥1 quit attempt were remarkably motivated to quit (80.8% versus 59%, *P*<0.001) (Table 2).

**Table 2:** Bivariate analysis taking the willingness to quit cigarette smoking as dependent variable.

**Factor**	**Low motivation** ** (n=210)**		**High motivation** **(n=156)**		***P*** ** value**
	**Number**	**Percent**	**Number**	**Percent**	
Residence					0.060
Beirut	56	26.7	43	27.6	
Mount Lebanon	79	37.6	74	47.4	
North	75	35.7	39	25.0	
Submission to smoke at work					0.060
No	116	55.2	101	64.7	
Yes	94	44.8	55	35.3	
Smoking work					0.060
≤1 person	106	50.5	94	60.3	
>1 person	104	49.5	62	39.7	
Wheezing					0.020
No	190	90.5	128	82.1	
Yes	20	9.5	28	17.9	
Fagerstrom					0.310
Low dependence	61	29.0	53	34.0	
High dependence	149	71.0	103	66.0	
Mondor scale					0.001
Low motivation	138	65.7	65	41.7	
High motivation	72	34.3	91	58.3	
Greater effect of shocking images ^a^					0.001
No	88	41.9	26	16.7	
Yes	122	58.1	130	83.3	
Curiosity warning ^b^					0.001
Strongly disagree	148	70.5	73	46.8	
Disagree	25	11.9	15	9.6	
Agree	22	10.5	18	11.5	
Strongly agree	15	7.1	50	32.1	
Stop smoking ^c^					0.001
No	176	83.8	109	69.9	
Yes	34	16.2	47	30.1	
Importance of health warnings ^d^					0.001
Strongly disagree	84	40.0	29	18.6	
Disagree	24	11.4	17	10.9	
Agree	28	13.3	16	10.3	
Strongly agree	74	35.2	94	60.3	
Real quit attempt duration					0.001
<1* month	139	66.2	69	44.2	
³1 month	71	33.8	87	55.8	

^a^ Greater effect of images: greater effect of shocking images on tobacco boxes than warning text ^b^ Curiosity warning: curiosity to ask a specialist for help in quitting ^c^Stop smoking: ever stopped smoking due to the warnings ^d^ Importance of health warnings: Importance of health warnings on cigarette boxes

The results of the bivariate analysis taking the intention to quit in 2 months as the dependent variable, showed that smokers living in Beirut and Mount Lebanon, as well as employed and having chronic wheezing had a higher intention to quit in 2 months (*P*<0.001, *P*=0.05 and *P*=0.05 respectively). In addition, highly motivated persons had significantly more intention to quit in 2 months (62.7% versus 33%, *P*<0.001), while this latter group revealed that they would change the favorite cigarette brand if the manufacturing company decides to use shocking images and consider shocking pictures have hypothetically greater effect than simple warning text currently used with (64.1% versus 40.5%, *P*<0.001) and (83.8% versus 56.9%, *P*<0.001) respectively. Evidently, people with an intention to quit in 2 months had significantly more quit attempts (83.3% versus 55.5%, *P*<0.001) (Table 3).

**Table 3:** Bivariate analysis of intention to quit smoking in 2 months as dependent variable

**Factors**	**No (n=200)**		**Yes (n=126)**		***P*** ** value**
	**Number**	**Percent**	**Number**	**Percent**	
Residence					0.001
Beirut	52	26.0	42	33.3	
Mount Lebanon	69	34.5	63	50.0	
North	79	39.5	21	16.7	
Submission to smoke at work			0.080
No	112	66.0	83	65.9	
Yes	88	44.0	43	34.1	
Work situation					0.050
Employed	136	68.0	99	78.6	
Unemployed	21	10.5	13	10.3	
Never employed	43	21.5	14	11.1	
Wheezing (yes)					0.050
No	180	90.0	104	82.5	
Yes	20	10.0	22	17.5	
Fagerstrom scale					0.750
Low dependence	62	31.0	37	29.4	
High dependence	138	69.0	89	70.6	
Number of cigarette			0.160
1-5 cigarettes	13	6.5	10	7.9	
6-10 cigarettes	23	11.5	6	4.8	
11-19 cigarettes	22	11.0	19	15.1	
≥20 cigarettes	142	71.0	91	72.2	
Age of smoking onset			0.050
10-14 yr	27	13.5	22	17.5	
15-17 yr	47	23.5	42	33.3	
≥18 yr	126	63.0	62	49.2	
Mondor scale					0.001
Low motivation	134	67.0	47	37.3	
High motivation	66	33.0	79	62.7	
Greater effect of images ^a^			0.001
No	89	44.5	28	22.2	
Yes	111	55.5	98	77.8	
Curiosity warning ^b^					0.001
Strongly disagree	122	62.6	78	66.7	
Strongly agree	18	9.2	25	21.4	
Agree	27	13.8	7	6.0	
Disagree	28	14.4	7	6.0	
Change cigarette brand ^c^			0.001
No	121	60.5	51	40.4	
Yes	79	40.5	75	59.5	
Importance of health warnings ^d^			0.001
Strongly disagree	82	42.1	23	19.7	
Strongly agree	53	27.1	82	70.1	
Agree	33	16.9	8	6.8	
Disagree	27	13.8	4	3.4	
Quit attempt					0.001
Yes (≥1 quit attempt)	111	55.5	105	83.3	

^a^ Greater effect of images: greater effect of shocking images on tobacco boxes than warning text ^b^ Curiosity warning: curiosity to ask a specialist for help in quitting ^c^Change cigarette brand: change cigarette brand if shocking images on cigarette box ^d^ Importance of health warnings: Importance of health warnings on cigarette boxes.

When taking the intention to quit smoking in 6 months as the dependent variable, the bivariate analysis results showed that people who smoked indoor and those with high motivation had a significantly higher intention to quit (83.3% versus 64%, *P*=0.040) and (60% versus 33%, *P*=0.004) respectively. Smokers with an intention to quit in 6 months consider shocking warnings have a hypothetically greater impact than simple warning text currently used on their behavior with (83.3% versus 56.9%, *P*=0.006). Clearly, people with an intention to quit in 6 months had significantly more quit attempts (83.3% versus 55.5%, *P*=0.004) (Table 4).

**Table 4:** Bivariate analysis of intention to quit smoking in 6 months as dependent variable

**Variables**	**No (n=200)**		**Yes (n=30)**		***P*** ** value**
	**Number**	**Percent**	**Number**	**Percent**	
Gender					0.010
Male	113	56.5	24	80.0	
Female	87	43.5	6	20.0	
Smoking indoor			0.040
No	72	36.0	3	16.7	
Yes	128	64.0	25	83.3	
Presence of respiratory diseases			0.060
No	173	86.5	22	73.3	
Yes	27	13.5	8	26.7	
Fagerstrom scale			0.910
Low dependence	62	31.0	9	30.0	
High dependence	138	69.0	21	70.0	
Mondor scale					0.004
Low motivation	134	67.0	12	40.0	
High motivation	66	33.0	18	60.0	
Stop smoking ^a^					0.100
No	166	83.0	21	70.0	
Yes	34	17.0	9	30.0	
Influenced by health warnings ^b^			0.001
No	177	88.5	18	60.0	
Yes	23	11.5	12	40.0	
Changed your smoking habits			0.001
No	186	93.0	22	73.3	
Yes	14	7.0	8	26.7	
Greater effect of images ^c^			0.006
No	89	44.5	5	16.7	
Yes	111	55.5	25	83.3	
Curiosity warning ^d^			0.001
Strongly disagree	127	62.6	9	30.1	
Strongly agree	18	9.2	19	63.3	
Agree	27	13.8	1	3.3	
Disagree	28	14.4	1	3.3	
Change cigarette brand ^e^			0.040
No	121	60.5	12	40.0	
Yes	79	39.5	18	60.0	
Importance of health warnings ^f^			0.001
Strongly disagree	82	42.1	3	10.0	
Strongly agree	53	27.2	20	66.7	
Agree	33	16.9	0	0.0	
Disagree	27	13.8	7	23.3	
Types of warning labels ^g^			0.070
Textual	13	6.7	2	6.7	
Graphic	99	50.8	18	60.0	
Both	37	19.0	9	30.0	
Quit attempt					0.004
No (<1 quit attempt)	89	44.5	5	16.7	
Yes (≥1 quit attempt)	111	55.5	25	83.3	

^a^Stop smoking: ever stopped smoking due to the warnings ^b^Influenced by health warnings: Influenced by the health warnings on cigarette packages ^c^Greater effect of images: greater effect of shocking images on tobacco boxes than warning text ^d^ Curiosity warning: curiosity to ask a specialist for help in quitting ^e^ Change cigarette brand: change cigarette brand if shocking images on cigarette box ^f^ Importance of health warnings: Importance of health warnings on cigarette boxes ^g^ Types of warning labels: Types of warning labels on cigarette packs more effective in quitting

The first logistic regression taking readiness to quit as the dependent variable showed that highly motivated smokers as shown by the Mondor scale and smokers having chronic wheezing would significantly have an increased readiness to quit by approximately 2 times (*P*=0.007, ORa=1.98; 95% CI: 1.21, 3.26 and *P*=0.020, ORa=2.35; 95% CI: 1.15, 4.81, respectively). In addition, smokers having a lot of curiosity to ask a specialist for help in quitting and having a real quit attempt duration of 1 month or more would significantly have more readiness to quit by 6.8 times and 2.15 times subsequently (*P*<0.001, ORa=6.8; 95% CI: 3.36, 13.74 and *P*=0.003, ORa=2.15; 95% CI 95%: 1.30, 3.54, respectively) (Table 5).

**Table 5:** Logistic regression taking the willingness to quit cigarette smoking as dependent variable

**Variables**	**Motivation**		**OR (95% CI)**
	**Low**	**High**	
Smoking at work			
≤1 person	106	94	1.00
>1 person	104	62	0.58 (0.35, 0.96)
Mondor scale			
Low motivation	138	65	1.00
High motivation	72	91	1.98 (1.21, 3.26)
Wheezing			
No	190	128	1.00
Yes	20	28	2.35 (1.15, 4.81)
Greater effect of images ^a^			
No	88	26	1.00
Yes	122	130	2.22 (1.22, 4.05)
Curiosity warning ^b^			
Strongly disagree	148	73	1.00
Disagree	25	15	1.32 (0.61, 2.88)
Agree	22	18	1.67( 0.77, 3.63)
Strongly agree	15	50	6.8 (3.36, 13.74)
Importance of health warnings ^c^			
Strongly disagree	84	29	1.00
Disagree	24	17	1.6 (0.67, 3.82)
Agree	28	16	1.28 (0.53, 3.07)
Strongly agree	74	94	2.15 (1.15, 4.04)
Real quit attempt duration			
<1 month	139	69	1.00
≥1 month	71	87	2.15 (1.30, 3.54)

^a^ Greater effect of images: If these shocking images were used on tobacco boxes, would they have greater effect than simple warning text currently used? ^b^ Curiosity warning: curiosity to ask a specialist for help in quitting ^c^ Importance of health warnings: Importance of health warnings on cigarette boxes

A second logistic regression was conducted taking the intention to quit in 2 months as the dependent variable. Smokers residing in Mount Lebanon and North Lebanon versus the ones residing in Beirut have significantly less intention to quit smoking in 2 months by 18% and 67% respectively (*P*=0.01, ORa=0.82; 95% CI: 0.40, 1.64 and *P*=0.010, ORa=0.33; 95% CI: 0.15, 0.75 respectively). Highly motivated smokers and smokers having chronic wheezing have a more intention to quit in 2 months by 83% and 2.27 times respectively (*P*=0.040, ORa=1.83; 95% CI: 1.03, 3.28 and *P*=0.050, ORa=2.27; 95% CI: 1.00, 5.15 respectively). Additionally, smokers who considered reporting health warnings on packages is crucial and who had past quit attempt had more intention to quit in 2 months by 4.41 and 4.39 times respectively (*P*<0.001, ORa=4.41, 95% CI: 2.26, 8.60 and *P*<0.001, ORa=4.39, 95% CI: 2.24, 8.64, respectively) (Table 6).

**Table 6:** Logistic regression taking the intention to quit smoking in 2 months as dependent variable

**Variables**	**Intention to quit**		**OR (95% CI)**
	**No**	**Yes**	
Residence			
Beirut	52	42	1.00
Mount Lebanon	69	63	0.82 (0.40, 1.64)
North	79	21	0.33 (0.15, 0.75)
Wheezing smokers			
No	180	104	1.00
Yes	20	22	2.27 (1.00, 5.15)
Mondor motivation scale			
Low motivation	134	47	1.00
High motivation	66	79	1.83 (1.03, 3.28)
Number of cigarettes per day			
≤5 cigarettes	13	10	1.00
6-10 cigarettes	23	6	0.36 (0.08, 1.59)
11-19 cigarettes	22	19	1.72 (0.46, 6.38)
≥20 cigarettes	142	91	1.30 (0.42, 3.98)
Importance of health warnings ^a^			
Strongly disagree	82	23	1.00
Disagree	27	4	0.45 (0.13, 1.61)
Agree	33	8	0.59 (0.26, 1.65)
Strongly agree	53	82	4.41 (2.26, 8.60)
Changing the favorite brand ^b^			
No	121	51	1.00
Yes	79	75	2.11 (1.17, 3.80)
Quit attempts			
No	89	21	1.00
Yes	111	105	4.39 (2.24, 8.64)

^a^ Importance of health warnings: Importance of health warnings on cigarette boxes ^b^Changing the favorite brand: If your favorite cigarette brand/company decide to change the look of its cigarette boxes with shocking images on smoking health damage, would you think of changing it?

A third logistic regression taking the intention to quit smoking in 6 months as dependent variable revealed that people who smoke indoor and the ones who have a declared disease by a physician would significantly have a more intention to quit in 6 months by more than 5 times and 4 times respectively (*P*=0.03, ORa=5.13; 95% CI: 1.17, 22.41 and *P*=0.05, ORa=4.27; 95% CI: 1.01, 18.05 respectively). In addition, smokers who were more influenced by the health warnings on packages (by a reduction of a daily number of cigarette smoked), had more intention to quit in 6 months by 4.7 times (*P*=0.02, ORa=4.73, 95% CI: 1.25, 17.90), while considering the report of warnings on cigarette boxes as being very important and having a lot of curiosity to seek help to quit would significantly increase the intention to quit in 6 months (*P*=0.010, ORa=9.38; 95% CI: 2.01, 43.55 and p<0.001, ORa=18.18, 95% CI: 5.02, 65.78 respectively). Moreover, having past quit attempts would increase the intention to quit in 6 months (*P*=0.007, ORa=7.48; 95% CI: 1.73, 32.43) (Table 7).

**Table 7:** Logistic regression taking the intention to quit in 6 months as dependent variable

**Variables**	**Intention to quit**		**OR (95% CI)**
	**No**	**Yes**	
Gender			
Male	113	24	1.00
Female	87	6	0.31 (0.08, 1.13)
Smoking inside the house			
No	72	5	1.00
Yes	128	25	5.13 (1.17, 22.41)
Disease declared by a doctor			
No	173	22	1.00
Yes	27	8	4.27 (1.01, 18.05)
Influenced by health warnings ^a^			
No	177	18	1.00
Yes	23	12	4.73 (1.25, 17.90)
Importance of health warnings ^b^			
Strongly disagree	82	3	1.00
Disagree	27	7	3.51 (0.56, 20.55)
Strongly agree	53	20	9.38 (2.01, 43.55)
Curiosity warning ^c^			
Strongly disagree	127	9	1.00
Disagree	28	1	0.28 (0.02, 3.07)
Agree	27	1	0.42 (0.04, 4.34)
Strongly agree	18	19	18.18 (5.02, 65.78)
Quit attempts			
No	89	5	1.00
Yes	111	25	7.48 (1.73, 32.43)

^a^Influenced by health warnings: Influenced by the health warnings on cigarette packages ^b^Importance of health warnings: Importance of health warnings on cigarette boxes ^c^Curiosity warning: curiosity to ask a specialist for help in quitting

## Discussion

Our results showed that smokers who are highly motivated to quit smoking, having one or less smoker at work, with chronic wheezing defined as (whistling sounds heard on expiration more than 2 years), who consider shocking pictorial warnings as more effective than textual ones already implemented on cigarettes packages in helping to reduce/stop smoking, who consider the health warnings on packs as very important, having past quit attempt during the last year and real quit attempts duration for 1 month or more, were all factors associated with the stages of readiness to quit. Previous studies showed that earnings ^26^, the level of education^26^, male gender ^27^, past quit attempts ^28^, having a longer duration of past quit attempts ^28^, having lower nicotine dependence^28^, worrying about future health ^28^ were all factors associated with quit intentions in cigarette smokers.

The association between smoking constraints and intention to quit is not well explored. Smokers who have one smoker or less at work showed a more readiness to quit, in line with the results of Farkas et al. ^29^, where restricting smoking was linked to an important impact on quitting attempts. The same authors also found that living in smoke-free homes and working in smoke-free workplaces had significant influences on cessation^29^. Another Korean study ^30^ showed that the intention to quit was associated with home smoking limitations but not with workplace smoking restrictions. This finding supports the fact that smoking prohibitions may increase smokers’ motivation to think about quitting and inspire them to attempt to quit ^29^, thus promoting smoking cessation. Any rule put into practice should be assessed regularly for reinforcement, because the influence of a new rule on smokers’ intentions to quit may be at its uttermost initially and then may lessen with time after its implementation ^31^.

Interestingly, health warning labels seem to guide upcoming quitting attempts mainly through their ability to affect beliefs and judgements about the dangers of smoking, which in turn help to promote awareness concerning the bad consequences of smoking on one’s health, leading to stronger intentions to quit. By making warning labels more prominent and appealing, they should have a greater chance to change behavior^32^. Our results consolidate previous results, with these warnings increasing the readiness to quit, and the intention to quit in 2 and 6 months respectively. Therefore, stronger anti-tobacco messages and shocking pictorial warnings about the health effects of tobacco consumption on tobacco packages may also further reinforce the users’ intention to quit tobacco.

Concerning the influence of chronic wheezing, smokers have more readiness to quit and a more intention to quit in 2 months versus non wheezers, in line with another study ^33^. Furthermore, patients who had a respiratory disease diagnosed by their doctor had more intention to quit in 6 months. Consequently, patients with respiratory diseases may be perfectly positioned to profit from interventions that control their respiratory symptoms and motivate them to quit.

Our results consolidate previous results where motivation, as measured by Mondor scale, has been associated with more readiness to quit and intention to quit in 2 months^34^.

Moreover, the findings that smokers from the capital Beirut had more intentions to quit than smokers who were residing outside the city, highlight the fact that the limited tobacco use prevention and cessation campaigns in the city are not reaching the target population to encourage them to try to quit, in contrast with another study ^35^. Our results were expected since lower socioeconomic status individuals have higher amounts of tobacco use, are less expected to successfully quit, and may also be less likely to intend or attempt to quit^36^.

Furthermore, surveyed smokers declared that the use of shocking images on the cigarette boxes would significantly increase the readiness to quit and intention to quit in the 2 months. These results are comparable to another study that showed anti-tobacco messages in the media, in restaurants, and in public transportation were predictors for intention to quit tobacco^37^.

Looking at the predictors of quit intention, sociodemographic factors such as age did not significantly predict intention to quit tobacco, which is consistent with the findings of previous researches ^37^. However, male gender was significantly associated with the intention to quit in 6 months in our study, in opposite to the results of these studies ^37^.

Smoking is currently responsible for a third of all cancer deaths in many western countries. Tobacco smoking plays a strong role in the etiology of oral cancer, and oral cancer risk can be reduced by controlling of tobacco smoking in different countries^38^. Furthermore, moderate and heavy smoking carry a higher risk of lung cancer in women than in men, and this difference does not seem to be explained by lung volume ^39^. Another study estimating the economic burden of major cancer due to smoking in Iran showed that smoking was responsible for 16.5% of cancer deaths, 17.2% of years of potential life lost and 21% of the cost of productivity^40^.

With all being said, additional efforts are suggested to be made by concerned authorities to set up awareness campaigns in order to increase alertness on dangers of cigarette smoking and dependence implement new laws to decrease cigarette smoking in public places and encourage these adolescents to embrace health-promoting conducts.

Our study has several limitations. This was a cross-sectional design and therefore, we were unable to draw causal associations with such a design. The total sample size is acceptable, withdrawn from 3 governorates in Lebanon, however, cannot be extrapolated to the whole population. The replication of this study in different settings and geographic locations would provide better generalizability of the results. A selection bias is still, however, possible because of the refusal rate. The use of a questionnaire in patients may not always be accurate: problems in question understanding, recall deficiency and over or under evaluating symptoms, which can lead to a possible information bias. In addition, we relied on each subject’s self-reported data, which might contain some potential sources of bias, such as selective memory (to remember or not remember experiences or events that occurred at some point in the past) or social desirability bias as a result of the tendency of smokers to base their answers on what they think is theoretically right not what they usually do.

## Conclusions

Findings of this study improved current knowledge about the intention to quit cigarette smoking. Significantly higher intentions to quit cigarette smoking were associated with a higher motivation and influenced by shocking images and health related warnings on tobacco boxes. We hope that our results will initiate public health educational programs and interventions to surge the intention to quit cigarette smoking as the first step of quitting.

## Acknowledgements

Special thanks to all persons who helped us in the data collection and entry.

## Conflict of interest statement

The authors have nothing to disclose.

## Funding

None.

## Highlights

- Higher stages of readiness to quit were associated with high motivation, having chronic wheezing and real quit attempt duration of 1 month or more.
- Having past quit attempts would increase the intention to quit in 6 months.
- Higher intentions to quit cigarette smoking were associated with a higher motivation.
- Higher intentions to quit was influenced by shocking images and health related warnings on tobacco boxes.
- Initiating public health educational programs and interventions to surge the intention to quit cigarette smoking are warranted.
